# Assessment of Pulp-Tooth Ratio Based on 3D Segmentation for Age Estimation for Forensic Purposes in a Senegalese Population

**DOI:** 10.7759/cureus.109325

**Published:** 2026-05-21

**Authors:** Sankoung M Soumboundou, Mamadou Lamine Ndiaye, Rokhaya Ndiaye, Babacar Toure

**Affiliations:** 1 Odontology, Cheikh Anta Diop University, Dakar, SEN; 2 Dentistry, Cheikh Anta Diop University, Dakar, SEN

**Keywords:** 3d segmentation, age estimation, canine, cone beam ct, pulp

## Abstract

Introduction: The advent of 3D imaging has created a significant opportunity to enhance the accuracy of age estimation. By utilizing 3D segmentation, it is now possible to evaluate the three-dimensional pulp volume as an indicator of age. This study aimed to estimate the age of Senegalese individuals through 3D canine segmentation.

Materials and methods: A total of 90 Cone Beam CT radiographs of Senegalese subjects, aged between 20 and 87 years, were analyzed. The methodology involved segmenting the hard tissues of the canine teeth, followed by the pulp tissues, and subsequently calculating the ratio (R) of pulp tissue volume to hard tissue volume to estimate the subjects' chronological age. Correlations between quantitative variables were assessed, with statistical significance set at p<0.05 for all tests conducted.

Results: The findings indicated that the mean volumes of dental tissues were greater in men compared to women. There was no statistically significant relationship between age and hard tissue volume (p<0.126), with a correlation coefficient of r=0.351. However, the correlation between age and pulp tissue volume was statistically significant (p<0.018), with a correlation coefficient of r=-0.637. Additionally, the regression analysis between age and the ratio was statistically significant (p<0.001). The regression equation for age versus the ratio is provided below: Y = -2257.98216x + 116.3490967 (where Y represents age and x represents the ratio R).

Conclusion: The 3D segmentation of canine tissues presents an innovative method for estimating age in adult Senegalese individuals.

## Introduction

Estimating age is a complex process that requires substantial examiner experience and the use of validated, reliable methodologies. In forensic odontology, age estimation methods primarily rely on analysis of dental development, particularly the mineralization sequences of dental germs [1,2]. In this context, several methods have been proposed, including those developed by Demirjian et al., which have been applied with varying degrees of success across populations [2].

In addition, several dental atlases, such as those by Schour and Massler, WITTS 18, and the London Atlas, are available and allow for relatively straightforward age determination [3-5]. Among these, the London Atlas is currently considered the gold standard [2]. It has been widely validated in numerous studies worldwide, demonstrating high accuracy [6,7]. However, these methods, which rely on dental maturation, are mainly applicable to children and young adults, as their reliability declines sharply after age 23. For adult age estimation, other dental indicators have been explored, including dental translucency, periodontal changes, occlusal wear, and reduction of pulp volume [8-10]. Drusini was the first to demonstrate a correlation between pulp volume reduction and chronological age [11]. This approach is based on the physiological process whereby secondary dentin is continuously deposited throughout life, leading to a progressive reduction in pulp chamber volume [12].

Numerous studies have confirmed a significant correlation between pulp volume reduction and age. Nevertheless, methodologies that rely on measurements from two-dimensional radiographic images have important limitations. In 2D radiography, measurement ratios are not strictly 1:1, which may lead to inaccurate estimates and potential overestimation of values [13].

The advent of three-dimensional imaging has improved the accuracy of age estimation. Three-dimensional segmentation provides an accurate representation of the complex spatial anatomy of teeth, enabling more precise and reproducible assessments. This approach relies on image-processing techniques to isolate and reconstruct teeth as three-dimensional models [14]. Using volumetric data derived from cone-beam computed tomography (CBCT), age can be estimated by analyzing age-related anatomical changes in dental structures [15].

Canines, in particular, are of special interest in age estimation because of their robust morphology, long roots, and relative resistance to wear and environmental factors [16].

The aim of the present study was therefore to assess age estimation based on three-dimensional segmentation of pulp volume.

## Materials and methods

This descriptive, analytical cross-sectional study was conducted in the Department of Dental Radiology at Cheikh Anta Diop University of Dakar. Cone-beam computed tomography (CBCT) images were retrospectively collected from the database of the Department of Radiology, Faculty of Medicine, University of Dakar. All CBCT examinations were performed using a Carestream CS 9600 system (Carestream Dental, New York, USA). Image acquisition was carried out using a cylindrical field of view of 16 × 12 cm (180 to 250 microns).

The sample size was calculated based on previously reported data by Emanuela Gualdi-Russo et al., indicating an 85% agreement among individuals aged ≤30 years [17].

n=(Z^2×p×(1-p))/d^2 (〖1.96〗^2×0.85×(1-0.85))/〖0.07〗^2 =100

Where n is the required sample size, Z is the standard normal deviate corresponding to a 95% confidence level (Z = 1.96), p (set at 0.85) is the proportion reported by Emanuela Gualdi-Russo et al., and d is the margin of error (set at 0.07).

The inclusion criteria for canines were as follows: absence of dental caries, restorations, artifacts caused by metallic restorative materials on adjacent teeth or orthodontic appliances, pulpal calcifications, dental anomalies, excessive occlusal wear, and severe periodontal disease.

Digital Imaging and Communications in Medicine (DICOM) datasets were imported into ITK-SNAP 3D image processing and reconstruction software (version 4.2.2) for segmentation and volumetric analysis.

This study was conducted in accordance with the ethical principles of the Declaration of Helsinki. Ethical approval was obtained from the relevant institutional review board. Due to the retrospective nature of the study, informed consent was obtained from patients at the time of imaging, and all data were anonymized prior to analysis.

Segmentation

**Figure 1:**
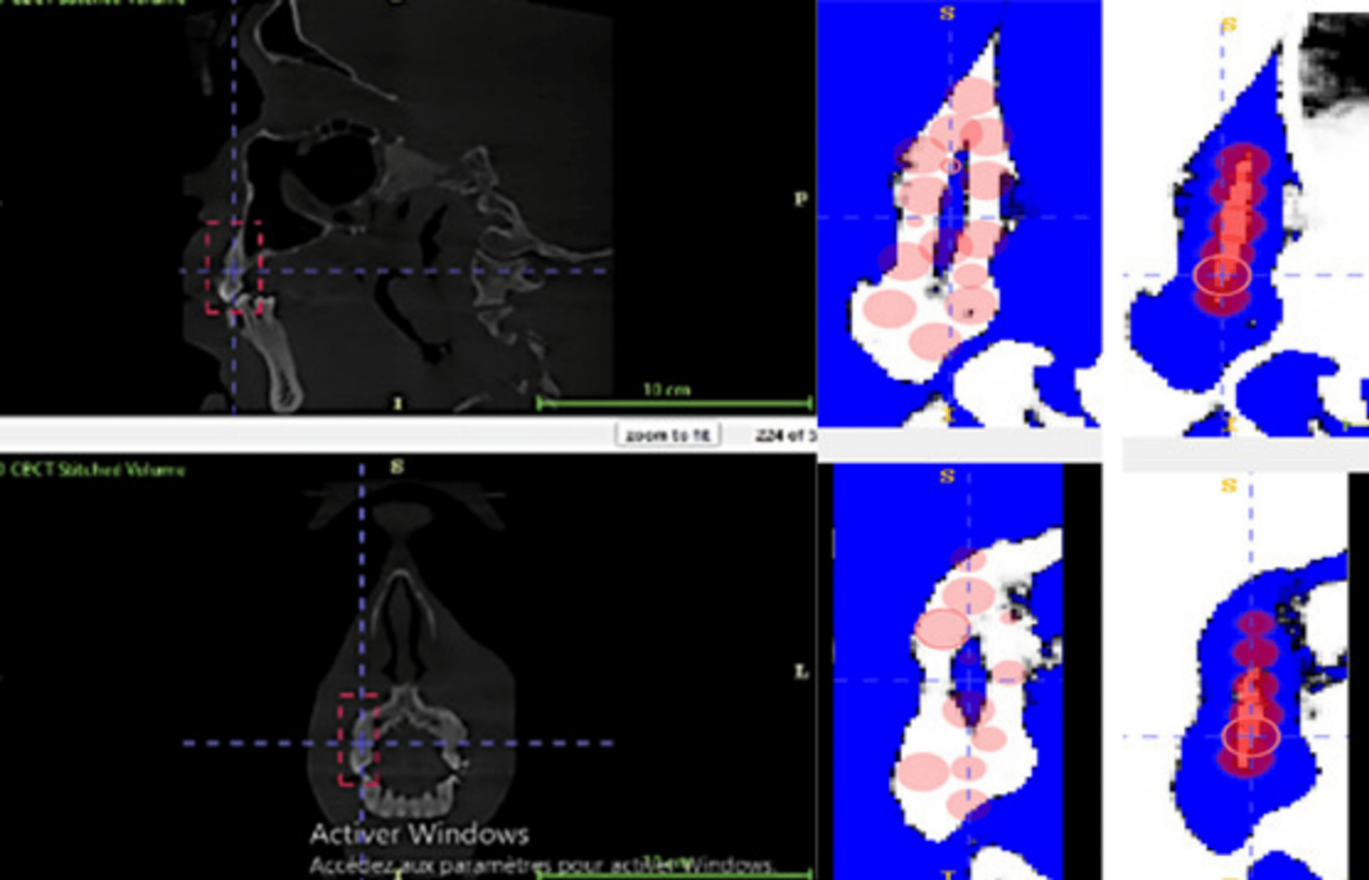
Image contrast enhancement followed by region of interest delineation and pre-segmentation

Segmentation of hard dental tissues and the pulp was performed in three main steps (Figure 1).

Segmentation procedure

Preprocessing

Image contrast was adjusted, and noise reduction was applied to enhance the visualization of anatomical structures.

Region of Interest (ROI) Delineation

The region of interest was carefully defined to isolate the target tooth and the surrounding structures.

Segmentation Initialization

Three-dimensional seed points (“bubbles”) were placed within the regions of interest across multiple planes.

**Figure 2:**
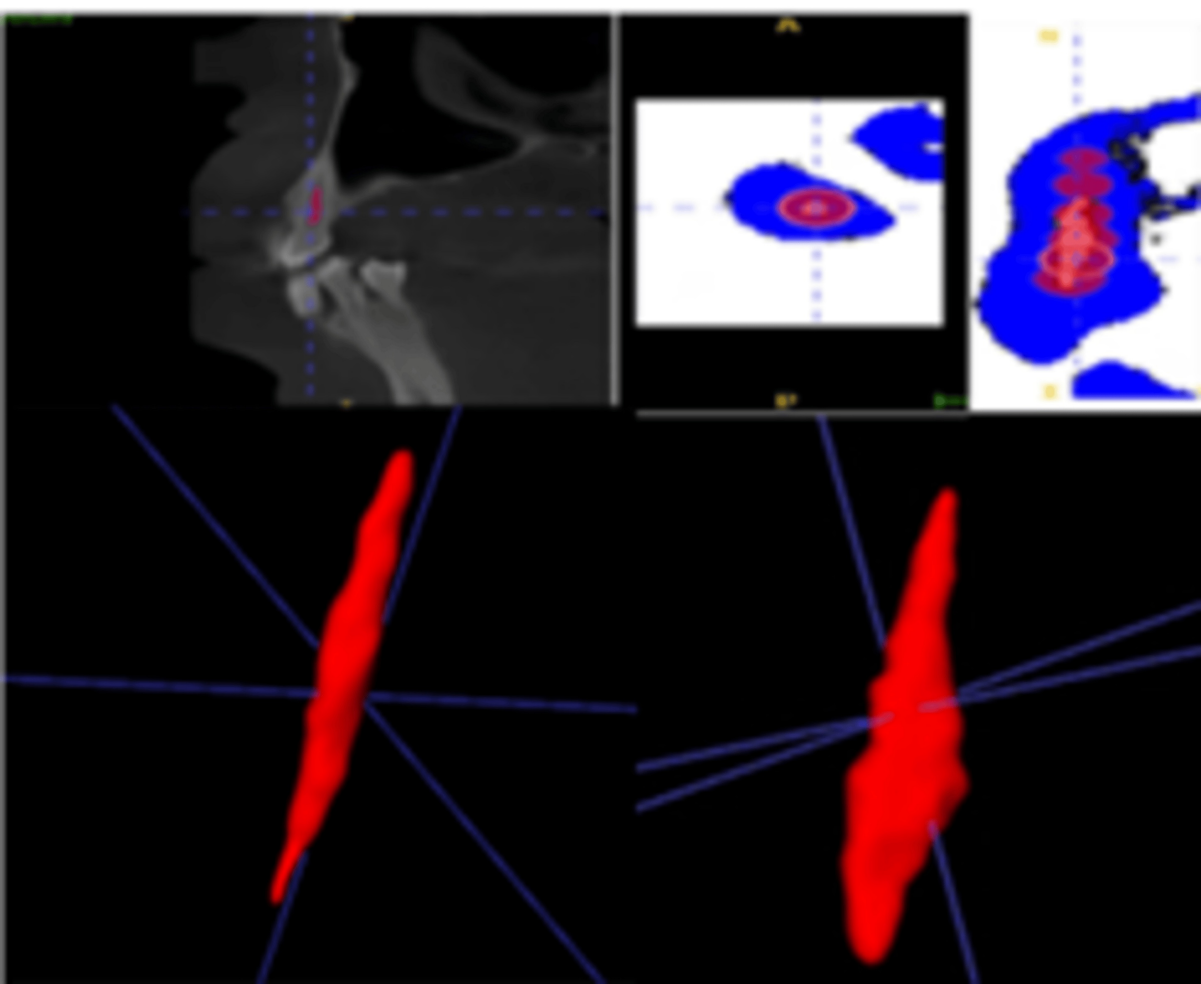
Pulp pre-segmentation and segmentation

Segmentation was considered complete when the 3D bubbles accurately delineated the contours of the pulp chamber (Figure 2).

By selecting the “Update” function, the segmentation was simultaneously displayed in the three orthogonal planes. The operator then refined the segmentation by scrolling through successive slices and adjusting the segmentation parameters as necessary.

Volumetric measurements

**Figure 3:**
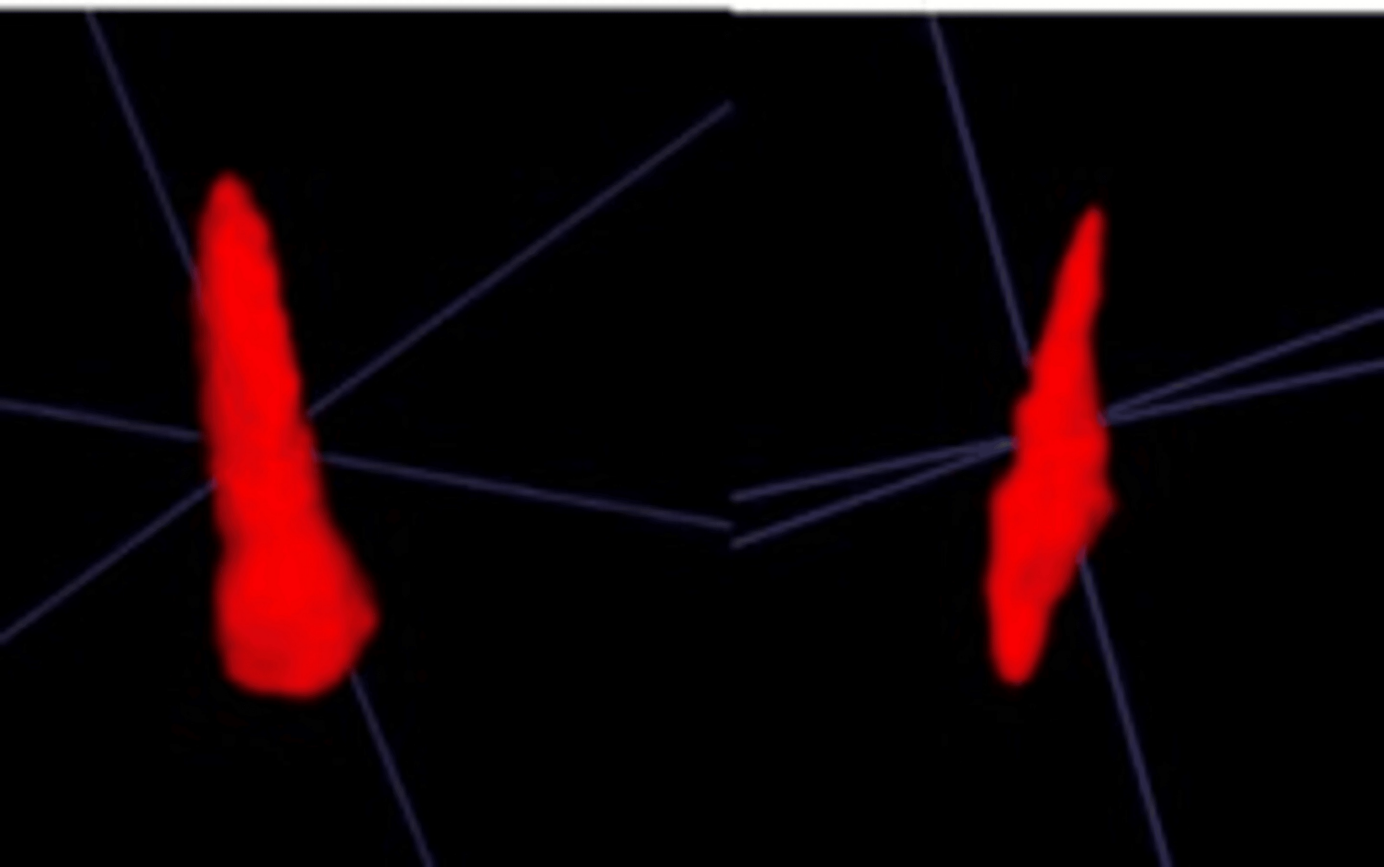
Three-dimensional segmented maxillary right canine

Once segmentation was complete (Figure 3), volumetric data were obtained by selecting the “Segmentation” menu and using the “Volumes and Statistics” function. For each segmented structure, the following parameters were recorded: a) number of voxels in the structure, b) volume of the structure (in cubic millimeters), and C) average image intensity within the structure.

The volume of each structure was calculated by multiplying the number of voxels by the volume of a single voxel. This step is essential for accurate quantification and highlights the importance of precise calculations to ensure reliability.

The pulp volume/tooth volume ratio was then determined using the following formula: PV/HTV (PTV = pulp volume; HTV = hard tooth tissue volume).

Statistical analysis

Statistical analyses were performed using JASP software (version 0.18.0). Descriptive statistics of the studied variables (dental pulp volume and hard dental tissue volume) were calculated and expressed as means ± standard deviations.

The correlation between dental pulp volume and chronological age was evaluated using Pearson’s correlation coefficient. A student’s t-test was applied to compare pulp volumes and hard dental tissue volumes between males and females.

A linear regression analysis was then conducted to assess the relationship between chronological age and the reduction in dental pulp volume, while accounting for the explanatory variables included in the model. A p-value < 0.05 was considered statistically significant.

## Results

Sociodemographic characteristics

A total of 90 cone-beam computed tomography (CBCT) scans from Senegalese subjects were analyzed, including 49 women (54.4%) and 41 men (45.6%). The mean age of the study population was 48.8 ± 17.5 years, ranging from 20 to 87 years.

Volumetric measurements of pulp, hard dental tissue, and pulp-to-tooth ratio

Table 1 presents the mean volumetric values obtained from the segmentation of hard dental tissues, pulp, and total tooth volume. The mean pulp-to-tooth volume ratio (R) was 0.030 ± 0.011, with values ranging from 0.009 to 0.067.

**Table 1:** Average volumes and ratios of canine dental tissues

volume (mm3)	Means-SD	Maximum	Minimum
Total Hard tissue volume (VDT)	638.36 ± 135.94	1215.11	395.79
Dentin volume (DV)	620.12 ± 134.05	1188	382.70
Pulp volume (PV)	18.23 ±6.19	34.94	5.56
Ratio	0.03 ±0.01	0.06	0.009

Table 2 shows the mean volumetric values of pulp and hard dental tissues stratified by sex. On average, both hard tissue and pulp volumes were higher in men than in women.

**Table 2:** Mean volumes of tissues stratified by sex

	Independent Samples T-Test					
Volume (mm3	Sex	n	Mean	SD	SE	p
Pulp (VP)	M	41	21.93	6.41	1.04	0.002**
	F	49	18.12	5.04	0.69	
Dentin (DV)	M	41	649.30	167.44	27.16	0.097
	F	49	599.48	114.64	15.89	
Tooth (Pulp and dentin)	M	41	671.23	168.66	27.36	0.078
	F	49	617.61	116.80	16.19	
Ratio	M	41	0.035	0.013	0.002	0.037*
	F	49	0.031	0.008	0.001	

**Figure 4:**
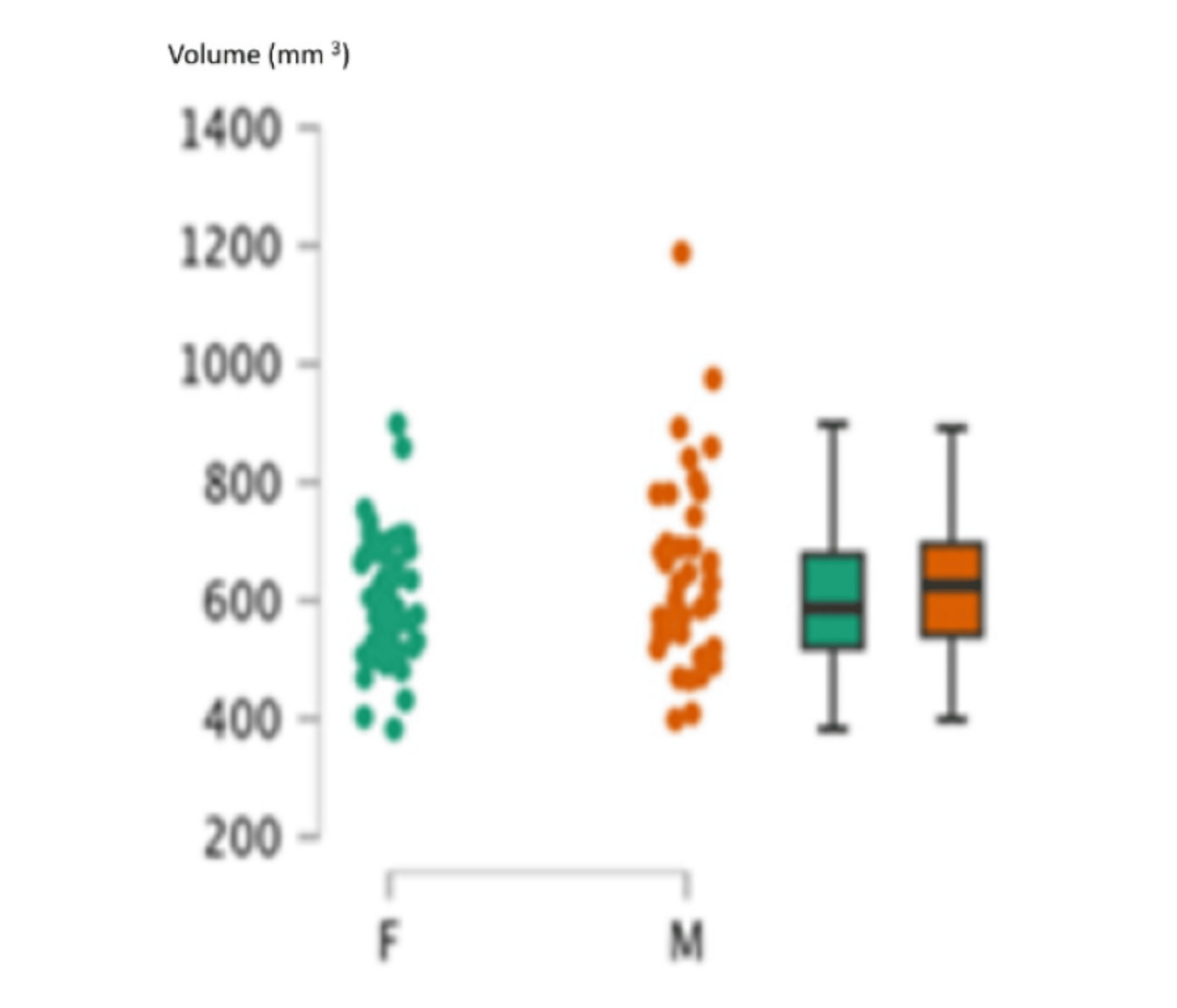
Means volume of hard tissues and pulp

Figure 4 illustrates the box-and-whisker plot of hard tissue volume according to sex.

**Figure 5:**
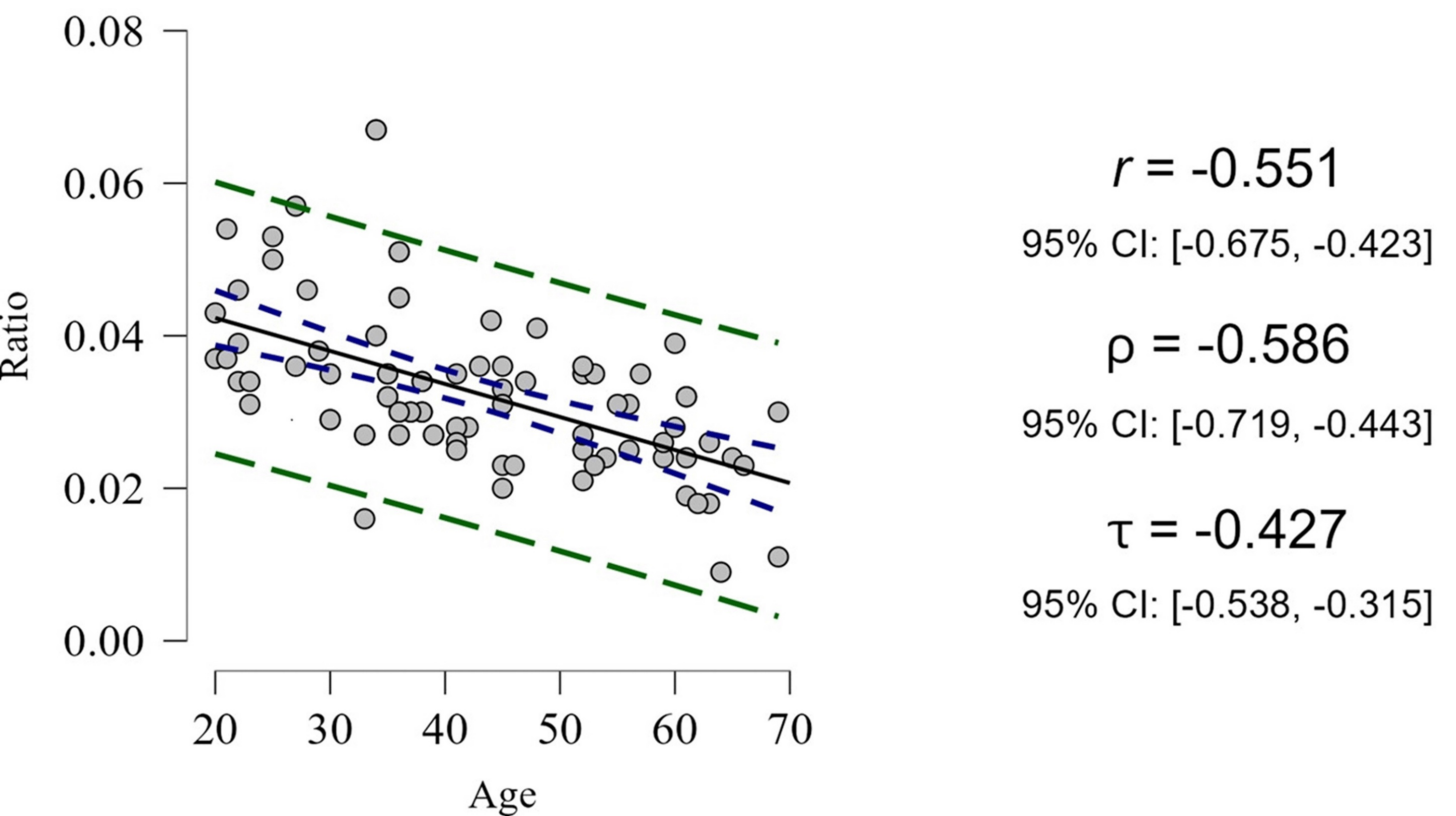
Correlation between age and ratio

Figure 5 reports the results of Pearson correlation analyses between chronological age and the mean volumes (hard tissue, pulp) and the R ratio.

**Figure 6:**
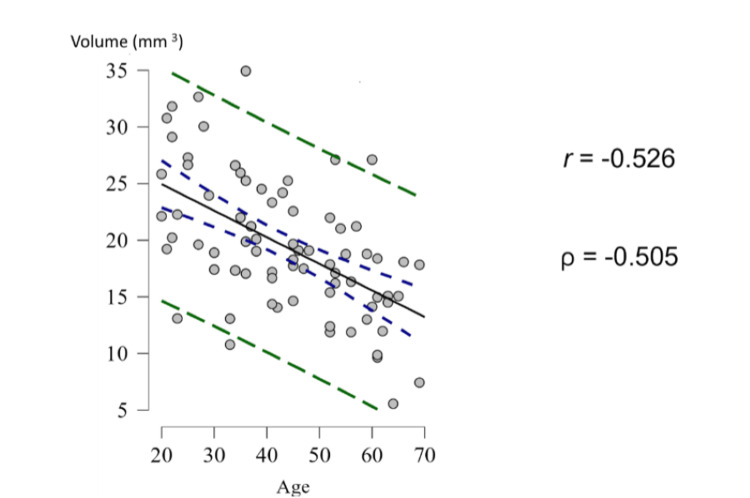
Correlation between pulp volume and age

A statistically significant correlation, r = -0.52 ( -0.66; -0.35), was observed between age and pulp volume (Figure 6).

Table 3 presents the correlation between chronological age and pulp volume using two statistical models (M0 and M1).

**Table 3:** Regression

	Regression between age and pulp volume				
Models	R	R^2^	R^2 ^_adjusted_	RMSE	p
Mo	0.000	0.000	0.000	13.313	0.001
M1	0.526	0.277	0.269	11.384	

Model M1

The correlation coefficient (R = 0.526) indicates a moderate positive correlation between pulp volume and age. The coefficient of determination (R² = 0.277) shows that 27.7% of the variability in age is explained by the model.

The adjusted R² = 0.269 confirms the model’s stability after adjustment. The RMSE = 11.384, which is lower than that of M0, indicates improved predictive accuracy.

The regression equation describing the relationship between age and the pulp-to-tooth volume ratio is y=65.16 -700.74* X.

**Figure 7:**
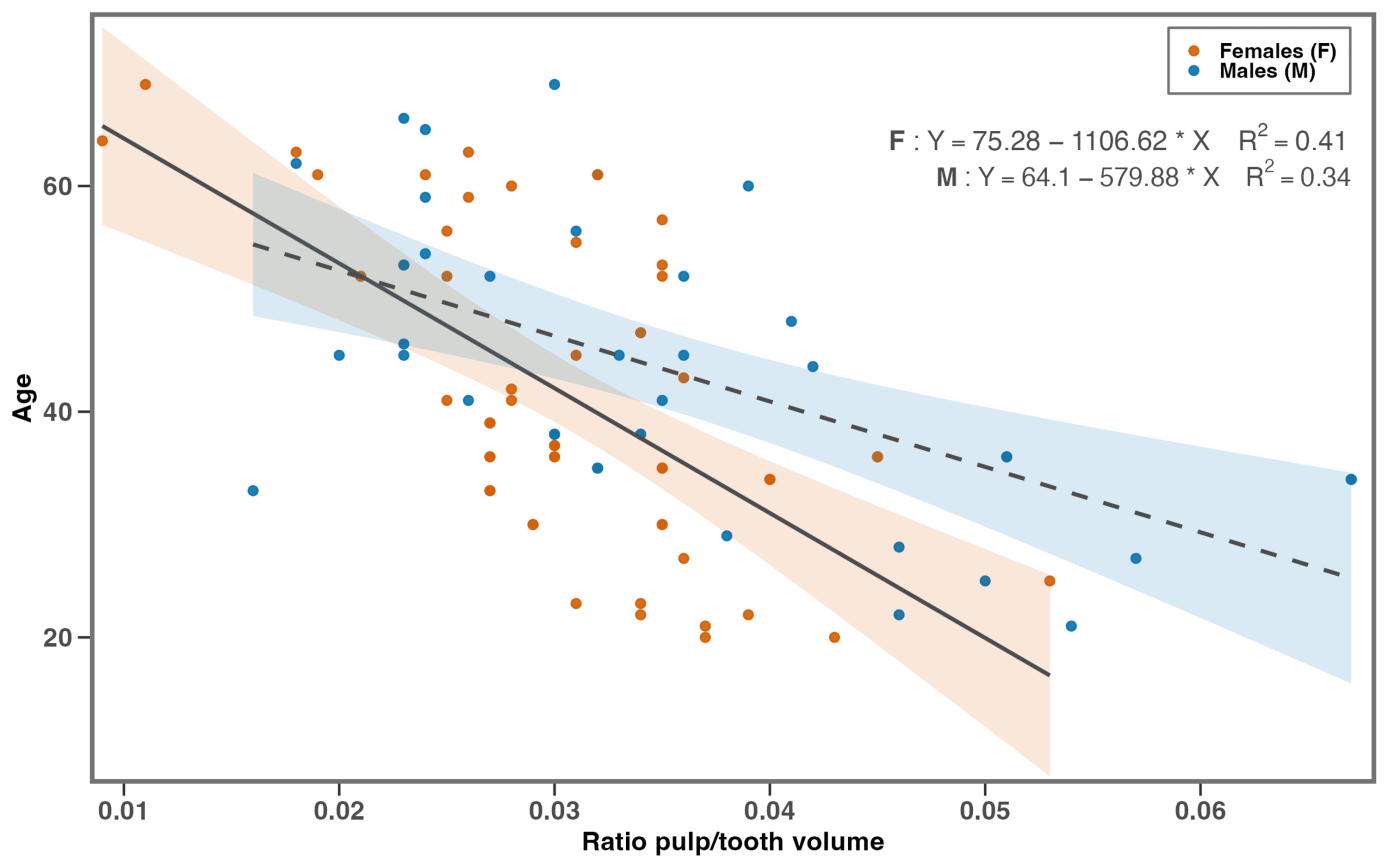
Linear regression

Figure 7 illustrates the regression analyses of age as a function of pulp volume, demonstrating a progressive decrease in pulp volume with increasing age. The regression between age and the pulp volume was statistically significant (p < 0.001).

Where X represents the pulp-to-tooth volume ratio (R).

## Discussion

Throughout an individual’s life, from birth to death, the dental organ undergoes two successive phases: an initial phase of growth and maturation, followed by a phase known as senescence or physiological aging. Using various indicators, these two phases have been considered in numerous studies to evaluate their relevance and accuracy in age estimation [18,19]. The aim of the present study was to estimate chronological age using three-dimensional segmentation of canines performed with ITK-SNAP software.

The proposed method involved semi-automatic segmentation of both pulp and dental hard tissues. The volumes of these structures were measured in cubic millimeters (mm³), and the pulp-to-hard tissue volume ratio (R) was subsequently calculated.

Research in forensic dentistry emphasizes the value of cone beam computed tomography (CBCT) due to its precision, cost-effectiveness, and mobility. CBCT is endorsed for post-mortem imaging and for juxtaposing antemortem and post-mortem records, considering bone and dental details. It delivers a detailed 3D visual of dental structures at an affordable cost [20].

Improved methods focus on single-root teeth and use software to reduce subjectivity and errors [21]. Most previous studies on age estimation based on structural changes in dental tissues relied on conventional two-dimensional imaging, such as periapical or panoramic radiographs. These include methods developed by Cameriere, Shiro Ito, Solheim, and Drusini, which are based on linear measurements or ratios, such as the relationship between pulp chamber height and crown height. However, linear measurements obtained from conventional radiographs may be subject to error or bias due to image distortion and projection limitations.

The end parts of roots and root canals are very small; measuring pulp volume in these areas is difficult and time-consuming, and the probability of measurement error is high. This is even more difficult in multirooted teeth. However, processes such as chewing and attrition over time cause secondary dentin deposition and a reduction in the volume of the crown and pulp of the tooth, while having no significant effect on the volume of the tooth root. Therefore, using the ratio of pulp chamber volume to crown volume to determine age may be preferable to other methods and has less error [18]. This was the reason for using the ratio of pulp chamber volume to crown volume as an age estimation index in the present study. Some studies used only pulp volume or pulp chamber volume to estimate the age, but Gulsahi et al., like the present study, used the ratio of pulp chamber volume to tooth crown volume. This ratio compensates for the errors that occur due to anatomical differences among different people [22].

In the present study, we sought to assess the accuracy of volumetric measurements obtained from teeth imaged using cone-beam computed tomography (CBCT), with a slice thickness ranging from 180 to 250 microns (Carestream system). Digital acquisition allowed segmentation across all axial sections of enamel, dentin, and pulp, enabling three-dimensional reconstruction and volumetric analysis rather than linear measurement. This approach provides a comprehensive representation of the entire tooth structure, overcoming the inherent limitations of two-dimensional analyses.

Pearson’s correlation analysis revealed no statistically significant correlation between age and hard tissue volume (r = 0.3). In contrast, a statistically significant correlation was observed between age and pulp tissue volume (r = 0.55). These findings are consistent with those reported by Rizky Merdietio Boedi et al. [15], who conducted a study on 99 individuals aged between 20 and 60 years and demonstrated a statistically significant correlation (R² = 0.6) between age and the ratio of pulp chamber volume to coronal dentin volume.

Similarly, in a Brazilian study using semi-automatic segmentation with ITK-SNAP, Souza et al. reported a strong negative correlation (r > −0.7) between age and the pulp-to-tooth volume ratio (Pv/Tv) [7].

Several studies have demonstrated that the strength of the correlation between dental volumes and age depends on the type of tooth analyzed. Ge Zhi-pu et al. reported the highest correlations in maxillary second molars [12]. Pinchi et al. investigated maxillary central incisors and observed a strong correlation between age and the pulp volume-to-total hard tissue volume ratio (p < 0.001) [19]. Jagannathan et al. focused on canines and also reported statistically significant results, reinforcing the relevance of this tooth type for age estimation [16].

Other biological indicators have also been explored. Emanuela Gualdi-Russo et al. demonstrated a correlation between age and dental cementum apposition (TCA) [17]. Variations between studies may be attributed to differences in age range and tooth selection. For example, Kumar et al. included subjects aged 10 to 70 and 13 to 73 years and obtained highly significant results [23].

Overall, the findings of the present study highlight a significant correlation between age and pulp volume, as well as between age and the pulp-to-hard tissue volume ratio. The semi-automatic segmentation approach used herein represents an innovative and promising method for age estimation. Nevertheless, the main limitation of this study lies in the semi-automatic nature of pulp segmentation using ITK-SNAP, which requires operator intervention and may introduce inter- or intra-observer variability [24,25].

A major strength of this study is the exclusive analysis of canines, which typically present a large pulp chamber. In contrast, volumetric measurement of pulp in roots and root canals of multi-rooted teeth remains challenging due to their small size [26,27].

The selection of canines is therefore particularly appropriate, as these teeth are less affected by masticatory forces and attrition, which contribute to secondary dentin deposition.

## Conclusions

In summary, the results of this study demonstrate a significant correlation between pulp volume and age, as well as between the R ratio and age. The semi-automatic segmentation technique employed represents an innovative approach that can be integrated with existing methods used for estimating the age of deceased individuals. This method is non-invasive and complies with the ethical requirements of a rigorous scientific framework. Sex does not appear to have a significant influence on the age regression equation or on the estimation process. Nevertheless, studies involving larger sample sizes would be beneficial to further validate and strengthen the reliability of this approach for age estimation.
